# Enhancing *Candida auris* diagnosis: a comprehensive evaluation of VIASURE *Candida auris* real-time PCR detection kit for rapid diagnostic responses

**DOI:** 10.1128/spectrum.03111-24

**Published:** 2025-05-19

**Authors:** Blanca Dehesa-García, Henar Alonso, María Paz Peris, Blanca Fortuño, Pilar Abad, Antonio Rezusta, Ana Milagro

**Affiliations:** 1 Health Research Institute Aragón 507423 , Zaragoza, Spain; 2 Department of Microbiology, Pediatrics, Radiology, and Public Health, Faculty of Medicine, Universidad de Zaragoza, Zaragoza, Spain; 3 Department of Animal Pathology, Faculty of Veterinary Sciences, Universidad de Zaragoza, Zaragoza, Spain; 4 Miguel Servet University Hospital, Microbiology, Zaragoza, Spain; Taichung Veterans General Hospital, Taichung, Taiwan

**Keywords:** *Candida auris*, qPCR, molecular diagnosis

## Abstract

*Candida auris*, a yeast that is highly resistant to antifungal treatments, represents a significant healthcare concern due to its rapid spread through surface contamination and patient colonization. A recent outbreak at the Miguel Servet University Hospital in Zaragoza, Spain, highlighted the urgent need for rapid diagnostic responses. The current 4-day duration for microbiological culture results hinders timely patient isolation and transmission prevention. This study aims to evaluate the VIASURE *Candida auris* Real-Time PCR Detection Kit (Certest Biotec) for diagnostic performance and analytical specificity compared to the standard methodology based on culture and MALDI-TOF MS characterization. From August 2023 to December 2023, the Microbiology Service of the Miguel Servet University Hospital processed 816 diagnostic samples. Accuracy testing resulted in adequate clinical validation values, as presented here: sensitivity, 0.98 (95% confidence interval [CI], 0.90 to 1); specificity, 1 (95% CI, 0.99 to 1); positive predictive value, 0.98 (95% CI, 0.90 to 1); and negative predictive value, 1 (95% CI, 0.99 to 1). The implementation of this molecular biology test in the laboratory promises to improve diagnostic efficiency by allowing rapid patient isolation, initiation of appropriate treatment, and effective isolation measures.

**IMPORTANCE:**

*Candida auris*, a resilient and antifungal-resistant yeast, poses a significant healthcare threat due to its rapid spread. A recent outbreak at a hospital in Zaragoza, Spain, emphasized the urgent need for faster diagnostics. The current four-day wait for culture results hampers timely patient isolation. This retrospective and comparative study demonstrates the favorable clinical parameters of a commercially available molecular diagnostic kit. The kit allows enhanced diagnostic efficiency, swifter patient isolation, and more effective control measures in the laboratory. In conclusion, the kit addresses the pressing challenges presented by *C. auris* in healthcare.

## INTRODUCTION

The recent inclusion of *Candida auris* in the World Health Organization’s inaugural fungal priority pathogen list underscores its critical threat to global public health (1). It has since been identified worldwide, across six continents, and in more than 40 countries (2). Antifungal drug resistance is a common feature of *C. auris* isolates, and invasive infections caused by multidrug-resistant *C. auris* have been associated with high mortality. Concerns have been raised regarding transmission, persistent colonization, and the need for effective disinfection measures (3, 4). Healthcare facilities have reported *C. auris* outbreaks in critically ill hospitalized patients with high crude mortality rates (30% to 72%) (5–7).

As with other nosocomial infections, but unlike other *Candida* species, *C. auris* appears to be highly transmissible between patients and in healthcare settings, including from contaminated environments or equipment. It is also associated with prolonged persistence in the environment (8, 9).

Outbreaks (*n* > 15 cases) of *C. auris* have been documented in Spain, the United Kingdom, Colombia, India, Pakistan, Panama, Russia, South Korea, Kenya, South Africa, Israel, the United States, and Venezuela (10–14). In addition to outbreak occurrences, it is noteworthy that *C. auris* has emerged as one of the primary *Candida* species implicated in candidemia, as indicated by several recent case series (15, 16). The recent *C. auris* outbreak identified at the Miguel Servet University Hospital (HUMS) has highlighted the need to find new alternatives to optimize diagnostic response times in the Microbiology Department. The objective of this project was to assess the clinical performance and analytical specificity of the VIASURE *Candida auris* Real-Time PCR Detection Kit (from now on referred to as “VIASURE assay”) using the standard procedure of the Clinical Microbiology Laboratory (CML) as the reference diagnostic method: microbiological culture followed by identification with matrix-assisted laser desorption ionization-time of flight mass spectrometry (MALDI-TOF MS). To comprehensively evaluate the VIASURE kit, the study also included the development of a specificity panel.

Through this dual approach of diagnostic accuracy and analytical specificity testing, the project aimed to provide robust evidence on the efficacy of the VIASURE *Candida auris* Real-Time PCR Detection Kit for both accurate clinical performance and shortened turnaround times compared to the current methods employed at CML.

## RESULTS

A total of 816 DNA samples were collected from 401 patients between August 30, 2023, and December 27, 2023, for the purpose of this study. Conventional culture method and matrix-assisted laser desorption ionization-time of flight mass spectrometry (MALDI-TOF MS) yielded 57/816 (6.99%) positive results and 759/816 (93.01%) negative results for *C. auris* infection. Turning our attention from the question of sample positivity to that of sample origin, out of the 17 clinical isolates analyzed, 15 reported a *C. auris-*positive result and 2 reported a *C. auris-*negative result, whereas out of the 799 specimens directly analyzed with VIASURE assay (non-isolates), 42 were *C. auris* positive and 757 were negative for *C. auris* detection.

Regarding the positive samples, they corresponded to 15 different patients, 11 of whom were male (73.3%) and 4 of whom were female (26.7%). With respect to the anatomical sampling locations from positive samples, 15/57 (26.3%) samples were clinical isolates. The clinical isolates were obtained from clinical samples, which included either double axillary and inguinal or triple epidemiological swab samples. A total of 42/57 (73.7%) positive samples were the result of direct analysis of the clinical sample, including double inguinal and axillary swab samples, triple epidemiological swab sample, an abscess sample, an epidemiological bronchoaspirate sample, and one urine sample. The VIASURE assay correctly identified 56/57 DNA samples that were positive for *C. auris*. The false-negative result corresponded to a triple epidemiological swab sample. No abnormal amplifications were observed, and all curves from positive *C. auris* samples exhibited a sigmoid shape with all typical phases of the real-time PCR curve: baseline, logarithmic, and plateau phases.

**Fig 1:**
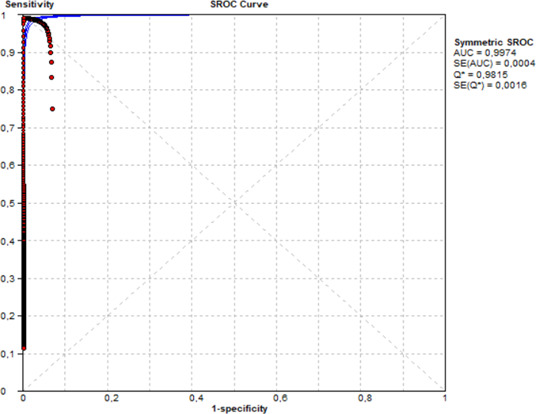
ROC curve analysis of VIASURE *Candida auris* Real-Time PCR Detection Kit (VIASURE assay) compared to culture and mass spectrometry identification (MALDI-TOF MS) for patient surveillance samples.

Accuracy testing demonstrated a high degree of concordance between the microbiological culture and mass spectrometry identification (current CML method) results and the VIASURE assay. A single false-negative result was obtained, which had a *Ct* value over the preestablished cutoff point (40.42 > 40) and was therefore interpreted as negative (Table 1). Subsequently, a receiver operating characteristic (ROC) curve analysis was performed on surveillance samples, and the obtained area under the curve score was 0.997. In addition, the calculated Youden index for the ROC curve was 0.981, and the cutoff value was <38.86. The calculated overall clinical sensitivity and specificity were 0.98 (95% CI [CI], 0.90 to 1) and 1 (95% CI, 0.99 to 1), respectively (Fig. 1).

**TABLE 1:** VIASURE *Candida auris* Real-Time PCR Detection Kit (VIASURE assay) compared to culture and mass spectrometry identification (MALDI-TOF MS)^*a*^

VIASURE assay	Culture and mass spectrometry identification					
	Positive	Negative	SE (95% CI)	SP (95% CI)	PPV (95% CI)	NPV (95% CI)
Positive	56	0	98.2 (90.6–100)	100 (99.5–100)	98.2 (90.7–100)	100 (99.5–100)
Negative	1	759				

^*a*^ SE, % sensitivity; SP, % specificity, PPV, % positive predictive value; NPV, negative predictive value; CI, confidence interval.

**Fig 2:**
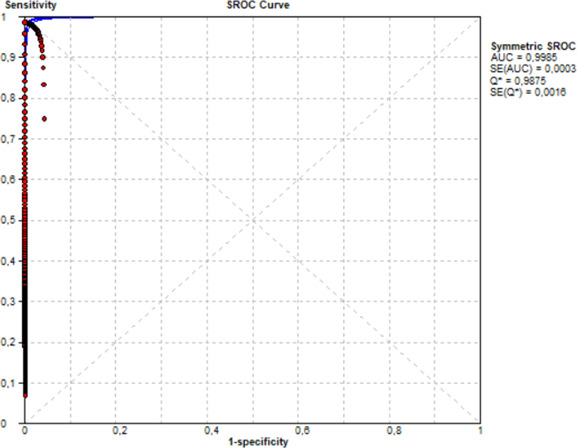
ROC curve analysis of VIASURE *Candida auris* Real-Time PCR Detection Kit (VIASURE assay) compared to culture and mass spectrometry identification (MALDI-TOF MS) for double inguinal and axillary swab samples.

The clinical performance assessment was performed over the 783 double inguinal and axillary swab samples, as they are the most common and abundant type of clinical specimen. This sample type was the only one to reach the minimal sample size calculation (*n* = 169) performed prior to the start of the study, as per the expected *C. auris* prevalence and the expected clinical sensitivity and specificity values according to the existing literature. Both the culture method with MALDI-TOF MS and the VIASURE assay yielded 34/783 (4.34%) positive and 749/783 (95.65%) negative results for *C. auris* infection using double inguinal and axillary swabs. The obtained clinical sensitivity and specificity were one (95% confidence interval [CI], 0.89 to 1) and one (95% CI, 0.99 to 1), respectively; and the positive predictive value and negative predictive value were one (95% CI, 0.89 to 1) and one (95% CI, 0.99 to 1) for double inguinal and axillary swabs (Table 2). A ROC curve analysis was performed on double inguinal and axillary swab samples, and the obtained area under the curve score was 0.998. In addition, the calculated Youden index for the ROC curve was 0.987, and the cutoff value was <38.86 (Fig. 2).

**TABLE 2:** VIASURE *Candida auris* Real-Time PCR Detection Kit (VIASURE assay) compared to culture and mass spectrometry identification (MALDI-TOF MS) using double inguinal and axillary swab samples^*a*^

VIASURE assay	Culture and mass spectrometry identification					
	Positive	Negative	SE (95% CI)	SP (95% CI)	PPV (95% CI)	NPV (95% CI)
Positive	34	0	100 (89.7–100)	100 (99.5–100)	100 (89.9–100)	100 (99.5–100)
Negative	0	749				

^*a*^ SE, % sensitivity; SP, % specificity, PPV, % positive predictive value; NPV, negative predictive value; CI, confidence interval.

Regarding the specificity panel results, the VIASURE assay correctly reported all samples as negative for *Candida auris* DNA. Therefore, the VIASURE assay did not report any false-positive results in the presence of *C. albicans*, *C. glabrata*, *C. lusitaniae*, *C. parapsilosis*, *Stenotrophomonas maltophilia*, *C. tropicalis*, or *Serratia marcescens* (23 samples).

## DISCUSSION

The overall (*n* = 816) sensitivity and specificity values obtained for the VIASURE assay, compared to MALDI-TOF MS results, were high and demonstrated the accuracy of the test. However, this analysis includes a range of sample types, and therefore, while it provides a comprehensive representation of the clinical context of routine testing and the reality of a clinical microbiology laboratory workflow during an outbreak, it is not correctly linked to a single sample type, as recommended for accurate clinical performance assessment.

The number of double inguinal and axillary swab samples included in the statistical calculations (*n* = 783) exceeded the number required according to the minimum sample size calculation (*n* = 169) (see Materials and Methods section). Consequently, the results obtained are considered representative. The obtained sensitivity and specificity values (1 [95% confidence intervals] [95% CI]: 0.89–1 and 1 [95% CI]: 0.99–1, respectively) compared to MALDI-TOF MS results not only show concordance with the routine diagnosis performed at the hospital but also align with similar validation studies of real-time PCR for *C. auris* (17–21). In addition, the ROC curve analysis determined the area under the curve score and cutoff value to be 0.998 and <38.86, respectively, showing an improvement in the test performance reported by Leach et al. (17), which had 0.940 and a cutoff value of <37.0.

In recent years, rapid testing methods have been developed to identify *C. auris* from patient samples (21). These include the utilization of selective media for the recovery of *C. auris* in culture (16), as well as the optimization of biochemical methods (https://www.cdc.gov/candida-auris/media/pdfs/testing-algorithm_by-method_508_1.pdf?CDC_AAref_Val=https://www.cdc.gov/fungal/candida-auris/pdf/Testing-algorithm_by-Method_508.pdf) and protein-based databases (http://www.cidrap.umn.edu/news-perspective/2018/04/fda-approves-rapid-diagnostic-test-candida-auris and https://www.rapidmicrobiology.com/news/new-fda-clearance-for-vitek-ms-expanded-id-for-challenging-pathogens) for *C. auris* identification.

Although these methods are excellent, they take considerable time (a few days to weeks) to confirm the identification of *C. auris* (6). Given the known fact that the minimum contact period for *C. auris* acquisition is 4 hours (13, 22), any method that takes longer leaves the clinicians in the dark for the management of individual patients and the ward as a whole.

Other rapid molecular-based assays using various real-time PCR chemistries have been developed (17–21, 23, 24). The targeted region for most of the molecular-based assays, including the one under study, is the internal transcribed spacer 2 (ITS2) region of the ribosomal gene. Once the high sensitivity and specificity values for *C. auris* identification were determined, the focus of the clinical validation studies of molecular assays has shifted towards the inclusion of epidemiological swab specimens, rather than solely clinical samples, as performed by Leach et al. (17), Sexton et al. (25), and Mulet Bayona et al. (19); the automation of the extraction step of the process, as considered by Lima et al. (24) and Leach et al. (18); or the elimination of the extraction step altogether to shorten the sample-to-result times, as done by Mulet Bayona et al. (19).

The validation of the assay under study was performed using a rapid automatic extraction method, as this step only increases the total diagnostic response time by 30 minutes while maintaining optimal detection sensitivity. Therefore, the turnaround time of the whole test was 2 hours, encompassing the duration of nucleic acid extraction and the PCR amplification protocol. Current clinical guidelines for *C. auris* management recommend testing newly admitted patients in the ward, as well as conducting weekly screening until discharge (22). The recommended sample types for *C. auris* colonization screening are axilla and groin swabs. However, other samples have also shown to be acceptable surveillance samples, including nares/oropharynx, ear, rectal, urine, and wound samples, which are associated with *C. auris* colonization (17, 22, 26, 27).

Previously published reports indicate that the sensitivity and specificity of molecular assays designed for *C. auris* identification using epidemiological and clinical swabs were high and similar across different clinical validation studies, with 89% clinical sensitivity (17), 93% sensitivity and 96% specificity (28), 96.6% sensitivity and 100% specificity (19), and 100% sensitivity and specificity (20), respectively.

The epidemiological context of this validation was that of an outbreak in the hospital ward, as described in the Spanish guidelines: “The onset of an outbreak should be suspected when there are at least two cases on the same ward, service or ward within one week or less, or a minimum of three cases within a maximum of one month” (26). Even with regular screening and following the recommended guidelines, Spain is reported to have a regional endemicity (29). These results are consistent with the context of *C. auris* introduction in Europe, which occurred first in the UK and in Spain (28). According to reports from the European Centre for Disease Prevention and Control, Spain has consistently reported *C. auris* colonization/infection cases since the first Spanish *C. auris* case in 2016, with a reduction in cases from 2017 to 2019. However, during and after the COVID-19 pandemic, *C. auris* cases rose again, following the trend observed across Europe (27, 30). The outbreak during which the clinical validation occurred took place from August 2023 to the end of December 2023. Out of 401 patients, 15 were identified with *C. auris-*positive results, yielding a positivity rate of 3.7%. This result is lower than the one employed as a reference for the sample size calculation (5.6%), which was the surveillance result from a Spanish hospital, although not the one where the present study took place, from 2017 to 2019 (22). This suggests a new trend toward the reduction in *C. auris* cases, likely due to improved control and management efforts, as well as the reduced ward burden after the end of the pandemic.

Finally, the limitations of the study must be assessed. The study was performed in the context of an outbreak at a single center, making the positive samples most likely derived from a single strain of origin. Furthermore, the comparative analysis was conducted using the routine methods of the hospital’s CML, which are culture and identification using MALDI-TOF MS, rather than a second molecular method, which would be an improved state-of-the-art comparative method. Moreover, in the present study, the specificity panel used to assess the analytical specificity of the assay was limited in size, and the species diversity was limited to other species previously identified in clinical samples from the routine testing of the CML. Although several sample types were included in the study, the performance of the VIASURE assay could only be fully studied in double inguinal and axillary swab specimens, as this was the only sample type with enough samples to perform a statistical analysis. For the remaining sample types included in the study, further analysis, including more samples, would be needed to validate the use of the VIASURE assay. Finally, to conclude the observation of a decrease in *C. auris* cases, further data from other Spanish hospitals would be needed to paint the whole picture of the current *C. auris* epidemiologic status nationwide.

### Conclusions

The VIASURE assay demonstrated high clinical sensitivity and specificity compared to the results of the reference routine method of the hospital, namely, culture on chromogenic agar and mass spectrometry identification using MALDI-TOF MS. Clinical sensitivity and specificity values were adequate according to the preestablished acceptance criteria for the analysis of double inguinal and axillary swab samples. All calculated values obtained from the statistical analysis of the data met the acceptance criteria, and no cross-reactivity was observed in the analytical specificity panel results.

This study demonstrated the usefulness of using the VIASURE *Candida auris* Real-Time PCR Detection Kit for diagnosing *C. auris* in double inguinal and axillary swab samples, meeting the expected clinical performance and analytical specificity values, and providing results within 2 hours. The shortened turnaround time compared to the current routine CML method may lead to an improvement in diagnosis and clinical management.

Furthermore, the analysis of complementary samples through both direct analysis of the clinical sample and analysis of the obtained clinical isolate showed preliminary satisfactory results, reporting a single false-negative result in a triple epidemiological swab sample with a Ct value over the preestablished cutoff point (40.42 > 40).

The findings obtained in this study lead to considering the implementation of rapid real-time PCR assays in clinical diagnostic laboratories, allowing high sensitivity and specificity values.

## MATERIALS AND METHODS

### Study design

This comparative study was performed at the CML of Miguel Servet University Hospital (Zaragoza, Spain), using DNA from a total of 401 patients suspected of *C. auris* infection. These samples were initially processed by the standard procedure of the clinical microbiology laboratory (CML), which was used as the reference technique: microbiological culture followed by identification with matrix-assisted laser desorption ionization-time of flight mass spectrometry (MALDI-TOF MS).

### Clinical samples

The minimum sample size was calculated using WinEpi 2.0 (http://www.winepi.net/winepi2/, accessed on 15th May 2024) (31), with the estimate proportion (random sampling and perfect diagnostic) option. The estimated proportion was 8.3% of the samples and 5.6% of the patients from the 2,107 outbreak in Valencia, Spain (22). The minimum sample size was determined to be 169 samples and 136 individuals, with an accepted error (or precision) of 5% and a confidence level of 95%.

This clinical study included a total of 816 specimens. Of these specimens, 783 were inguinal and axillary double swab specimens; one was a urine sample; 12 were nasal, pharyngeal, and rectal triple swab specimens; one was ascitic fluid; one was an abscess; one was an epidemiological bronchoaspirate; and 17 were clinical isolates. The clinical isolates were obtained from clinical samples, either double axillary and inguinal swab samples (11 samples) or triple epidemiological swab samples (six samples).

Culture and MALDI-TOF MS identification were performed during routine testing from August 30, 2023, to December 27, 2023, while DNA extractions and VIASURE assay analyses were conducted from October 5, 2023, to December 28, 2023. During the period in which VIASURE assay analysis was conducted, samples were stored at −20°C for a minimum of 11 hours and a maximum of 32 days. The utilization of stored samples, as opposed to fresh samples, is driven by the need to obtain approval for research use once the diagnostic use of the patient has been completed. Therefore, consistency in both *C. auris* and endogenous internal control results among the sample pool was confirmed prior to analysis, even with time-to-test variations.

### Specificity panel

This panel was designed to assess the analytical specificity of the assay in distinguishing *C. auris* from other related non-*C. auris Candida* species. By introducing a variety of related species into the specificity panel, the study aimed to ensure that the detection kit could reliably identify *C. auris* without cross-reactivity or false positives, which is critical in a clinical setting where multiple *Candida* species may be present. The analytical specificity of the real-time PCR assay was validated on a panel of 23 samples (15 direct clinical samples and eight clinical isolates from those samples), pulled from the routine testing results of the CML. These samples were from either epidemiological surveillance or clinical suspicion compatible with *C. auris* but were ultimately positive for other species (three *C*. *albicans*, four *C*. *glabrata*, one *C*. *lusitaniae*, one *C*. *parapsilosis*, three *Stenotrophomonas maltophilia*, two *C*. *tropicalis,* and one *Serratia marcenses*). This analysis was performed in a blinded manner, integrating these samples into the clinical samples group workflow.

Of these specimens, one was a bronchoaspirate (and its clinical isolate); two were catheter samples (and their clinical isolates); one was an inguinal and axillary double swab specimen; one was a pharyngeal swab sample; four were nasal, pharyngeal, and rectal triple swab specimens; one was a urine sample; one was a rectal swab sample (and its clinical isolate); and four were blood samples (and their clinical isolates).

Culture and MALDI-TOF MS identification were performed during routine testing between 2016 and 2023, while DNA extractions and VIASURE analyses were conducted from October 13, 2023, to December 19, 2023.

### Routine reference diagnosis

In the laboratory of Microbiology Service, the analysis is conducted using the conventional culture method. The media in which a sample is grown depends on the suspicion or the type of swab sample (Sabouraud agar, CHROMagar *Candida*, and fluconazole 32 ug/m chromogenic plates). Agar plates are incubated at 35–37°C for 24 to 48 h, and the identification of *C. auris* is carried out using matrix-assisted laser desorption ionization-time of flight mass spectrometry (MALDI-TOF MS) on the colonies that have grown. Negative results are reported after four days of incubation.

### Sample preparation for nucleic acid amplification test (NAAT) analysis

After seeding, the remnant of the samples was stored at −20°C until use for NAAT analysis. In the case of clinical isolates, they were processed on the same day the colonies were obtained and identified using MALDI-TOF MS. For clinical isolates, a loopful of isolated colonies was homogenized in 400 µL of molecular-grade water. Swab samples and urine samples preanalytical preparation is minimal and therefore referred to as “direct samples”. For the different swab samples, an aliquot of 200 µL resuspended in liquid transport media was taken for nucleic acid extraction, and for the urine sample, an aliquot of 200 µL was taken for nucleic acid extraction.

For NAAT analysis, both types of specimens were brought to room temperature, and an aliquot of 200 µL was taken. Once all the samples were prepared, the nucleic acids were extracted. DNA extractions were performed using 200 µL of the prepared clinical samples with the validated commercial kit MagDEA Dx reagent (batch: 46M040) and the automated MagLEAD 12gC instrument (Precision System Science), following the Protocol IC Card Magtration MagDEA Dx SV 200 Ver 1.0. All nucleic acid elutions were performed in 100 µL of elution buffer.

### Molecular method under evaluation

VIASURE *Candida auris* Real-Time PCR Detection Kit was designed for the specific detection and amplification of the ITS2 gene from *C. auris* using specific primers and fluorescent-labeled probes. *C. auris* was amplified using the FAM fluorophore, and the internal control was amplified with the HEX fluorophore. Each run incorporated a positive and a negative control, both provided in the kit. Each reaction was executed in a final volume of 20 µL, containing 15 µL of Master Mix and 5 µL of DNA template.

The VIASURE system RT PCR VS-CAU1XL-Exp786B (expiry date: 09/2025) was performed using the CFX96 real-time PCR system (Bio-Rad Laboratories, France), and the fluorescence threshold adjustment was automatically performed by the CFX Manager software. qPCR cycling conditions were as follows: polymerase activation at 95°C for two minutes, followed by 45 cycles with the denaturing step at 95°C for 60  seconds, and annealing and elongation steps at 60°C for 10  seconds.

### Data collection and analysis

The data were collected in an Excel file, including the results from the conventional culture method and VIASURE. The results of the conventional sowing method were considered the reference assays to calculate clinical sensitivity, specificity, and negative and positive predictive values (with 95% CI) using the MetaDisc v1.4 freeware software (32). A receiver operating characteristic (ROC) curve analysis was generated using MetaDisc v1.4 freeware software (32), and the area under the curve (AUC) score for the VIASURE assay results was calculated. The optimal diagnostic cutoff value was determined by calculating the Youden index of the ROC curve. The differences were considered statistically significant when the *P*-values were ≤0.05.

### Analysis result acceptance criteria

The acceptance criterion for clinical sensitivity, specificity, positive predictive value (PPV), and negative predictive value (NPV) is a calculated value of at least 80%, ideally reaching 95%. Regarding the receiver operating characteristic (ROC) curve analysis, the area under the curve (AUC) score and Q* index values should be close to 1, near the ideal test, with a calculated value of at least 0.95.

Regarding the analytical specificity panel, the acceptance criterion is to obtain no amplification for the *C. auris* target channel in any of the samples of other non-*C*. *auris Candida* species.
